# Comparison of microRNA expression in hippocampus and the marginal division (MrD) of the neostriatum in rats

**DOI:** 10.1186/1423-0127-20-9

**Published:** 2013-02-20

**Authors:** Si Yun Shu, Di Qing, Bin Wang, Qi-yi Zeng, Yan-chen Chen, Ying Jin, Chang-chun Zeng, Rong Bao

**Affiliations:** 1 Center of Pediatrics, Zhujiang Hospital, Southern Medical University, 510280, Guangzhou, Guangdong, China; 2 The College of Biophotonics, South China Normal University, 510631, Guangzhou, Guangdong, China; 3 Department of Pediatrics, Sun Yet-San Hospital, Zhong-Shan University, 510120, Guangzhou, China

**Keywords:** mRNA transcript, Oligo-microarray, Gene silence, Synapse formation, Learning and memory

## Abstract

**Background:**

MicroRNAs (miRNAs), a class of highly conserved small non-coding RNA molecules, are known to play essential roles in central nervous system (CNS) by causing post-transcriptional gene silencing. There is much evidence that miRNAs have specific temporal and spatial expression patterns in the mammal brain, but little is known about the role of the region specificity for the gene regulatory networks of the brain. This study represents the first attempt to perform a profiling analysis of the differential expression of miRNAs between hippocampus and the Marginal division (MrD) of the neostriatum in the rat brain.

**Results:**

Microarray was used to detect the expression of 357 miRNAs in hippocampus and the MrD from three rats. A short-list of the most dysregulated 30 miRNAs per rat was generated for data analysis, and the miRNAs that were represented in two or three short-lists were then further analyzed. Quantitative real-time reverse transcription-polymerase chain reaction (RT-PCR) was employed to validate the aberrantly expressed miRNAs obtained from the miRNA microarray analysis. A family of 11 miRNAs demonstrated differential expression between the MrD and hippocampus in more than one rat. Amongst these, miR-383 was differentially expressed in all three rats and up-regulated to the largest degree in rat one, and the ten other miRNAs, let-7d*, miR-181b, miR-187, miR-195, miR-214, miR-382, miR-411, miR-466b, miR-592 and miR-1224 were differentially expressed in at least two rats. Of these ten, besides miR-382 and miR-411 which were up-regulated in one rat and down-regulated in another, the other eight miRNAs retained a uniform direction of regulation (up-regulation or down-regulation) between different specimens. When further examined by RT-PCR, the aberrantly expressed miRNAs, except miR-383 and let-7d*, demonstrated differential expression that significantly correlated with the microarray findings.

**Conclusion:**

This study reported that the miRNA expression patterns in MrD was distinct from that of Hip, suggesting the role of miRNAs in the learning and memory function of the MrD probably different from hippocampus.

## Background

In 1993, Lee etc. firstly found miRNAs in the nematode *C. elegans* to be key regulators of developmental transitions [1], and since then miRNAs have been identified in species ranging from plants to humans [2,3]. It has been shown that miRNAs regulate expression of 30% or more of animal genes [4]. The first form of MiRNAs were long primary transcripts (pri-miRNAs), which are spilt into approximately 70 nt stem-loop precursors (pre-miRNAs) and then further processed to mature miRNAs in the cytoplasm by the RNaseIII Dicer [5]. MiRNAs, which are approximately 19–23 nucleotides in length, are post-transcriptional regulators that bind to complementary sequences in 3^′^-UTRs (Untranslated regions) of target mRNA transcripts, usually resulting in gene silencing [6,7]. Upon miRNA binding, mRNAs are then localized to the processing bodies (P-bodies), where they are either deadenylated and degraded or translationally inhibited [8]. The extent of complementarity between miRNAs and their targets may influence whether transcripts are degraded [3]. MiRNAs are expressed in a number of cell types at differing levels, and are especially abundant in the central nervous system, suggesting that they might be particularly important there [9].

The function of miRNAs in nervous system development has been identified initially through classical forward genetic approaches [10]. Recent evidence points to a widespread role for neural miRNAs at various stages of synaptic development, including dendritogenesis, synapse formation and synapse maturation [11]. It is well known that synapses mediate communication between nerve cells, and contribute to learning and memory. Besides synaptic function, the proposed roles of miRNAs in the vertebrate CNS may include neurogenesis [12], regulation of morphogenesis [13], dendrite formation [14], and silencing of non-neural mRNAs [15]. In another paper, miRNAs were shown to be involved in memory [16]. Furthermore, a number of studies indicated that miRNAs might contribute to the control of synapse function and plasticity in the adult [17]. All of these functions of miRNAs indicated that it plays an important role in mediating regulation of mRNA expression and function by changes in neuronal activity. Dendritogenesis, synapse formation and new protein synthesis have long been recognized critical for formation of long-term memories (LTMs) [18]. Additionally, miRNAs were found as biomarkers for cancer and other disorders [19], and are involved in the etiology of several brain disorders, including Parkinson’s disease [20], Alzheimer’s disease [21], and depression [22].

The MrD is a pan-shaped subdivision in the caudal border of the neostriatum surrounding the rostral edge of the globus pallidus [23]. The MrD was first discovered in the brains of rat, and then it was verified that it is a universal structure in the neostriatum of the mammalian rain, including rat, cat, monkey and humans [24]. The MrD consists of spindle-shaped neurons, with high level expression of certain neuropeptides, monoamines and their receptors, such as substance P, dynorphin B, neurokinin 1 receptor in the fibers, terminals and neuronal somata in the MrD by immunohistochemical and patch clamp methods [25-27]. The MrD was found to be involved in learning and memory by double-blind studies of Y-maze learning and long-term potentiation in rats [28]. The MrD is a new component of the limbic system and is a key linking region between the limbic system and the basal nucleus of Meynert. Functional magnetic resonance image (fMRI) studies illustrated that the MrD and the prefrontal cortex are involved in digital working memory in the human brain [29]. The MrD has been shown to contribute to associative learning and declarative memory by behavioral study in rats and by fMRI study in humans. Lesions in the MrD influenced the learning and memory function of the basal nucleus of Meynert and attenuated hippocampal long-term potentiation [30].

The hippocampus is a well established learning and memory-related area of brain, that by comparison to the MrD, has distinct biological functions. However some associations between the hip and the MrD are also found in many papers. Although the functional consequences of region-special expression of miRNAs is not yet known, we believe that the region-specific expression pattern may contribute to functional differences between the brain regions. The purpose of the current work is to investigate the miRNA expression in the MrD by comparison to that of in hippocampus by using microarray technology. The results of this study may be valuable to explain the mechanisms of learning and memory function of the MrD, and regulatory neural networks and will enable novel functional genomic analyses in the rat.

## Methods

### Animals and total RNA extraction

**Figure 1:**
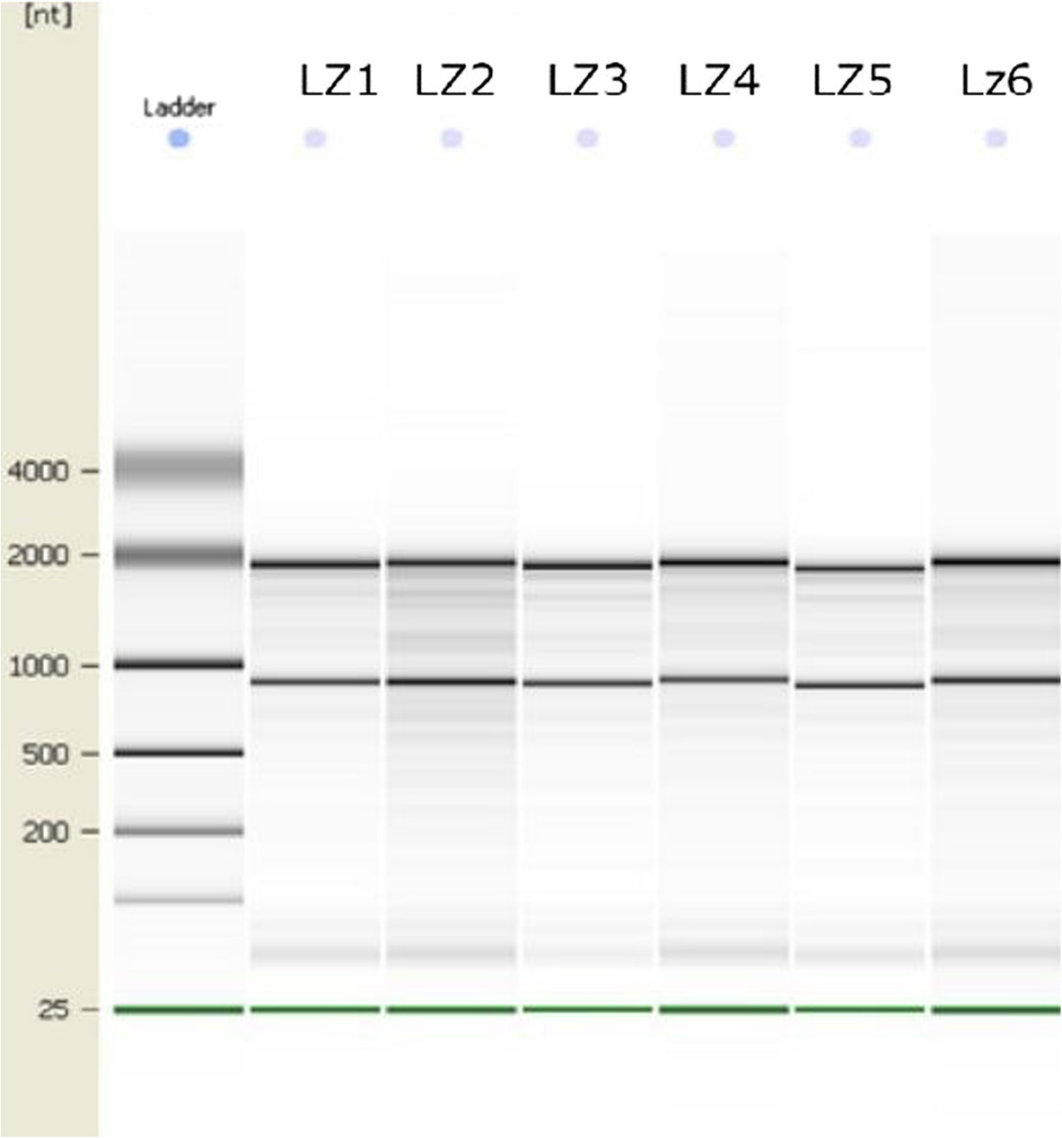
The electropherogram of the samples.

Adult male Sprague-Dawley rats (N=3), weighing about 220 g, were used in this study. Food and water was available ad libitum, and rats were kept on a 12-h lightdark schedule (light period 6 am-6 pm). The rats were deeply anesthetized with 10% chloral hydrate (3.5 ml/kg, i.p.) and then perfused, via the aorta, with 0.9% saline solution followed by cold 4% paraformaldehyde. The brains were removed in 4°C 0.9% saline solution. The tissue samples of Hip and MrD in brain were cut about 1 mm^3^ respectively (Table 1). Total RNA populations were extracted from brain tissue using the miRNeasy Mini Kit (Qiagen GmbH, Hilden, Germany) and were subjected to undergone a quality analysis to determine the quality and the quantity of the sample RNA (Table 1). The quality control was carried out with the Agilent 2100 Bioalyzer, using the RNA 6000 Nano Kit according the manufacturer’s recommendations. Within the resulting electropherogram, high quality RNA were characterized by two distinct bands, representing the 18 and 28 s rRNA (Figure 1:The electrophoretic patterns of RNA samples). All the samples were stored at -70°C for detecting miRNA expression profile.

**Table 1:** The information of samples and RNA quality check

**Sample**	**Description**	**Source**	**OD260/280**	RIN*
LZ1	Hip 1	hippocampus (Rat 1)	1.99	8.6
LZ2	MrD 1	Marginal division (Rat 1)	1.99	7.8
LZ3	Hip 2	hippocampus (Rat 2)	2.08	8.7
LZ4	MrD 2	Marginal division (Rat 2)	1.93	8.4
LZ5	Hip 3	hippocampus (Rat 3)	2.02	8.8
LZ6	MrD 3	Marginal division (Rat3)	1.96	8.4

*RIN: RNA integrity number.

### miRNA microarray screening

Febits biochip “Geniom Biochip MPEA *rattus norvegicus*” was used for this analysis. These probes are designed as the reverse complements of all major mature miRNAs and the mature sequences as published in the current Sanger miRBase release (version 14.0 September 2009, see http://microran.sanger.ac.uk/seqences/index.shtml) [31] for *rattus norvegicus*.

Additional nucleotides are bound on the 5^′^end of each capture oligonucleotide necessary for the enzymatic extention in the labeling procedure. The probes are synthesized with intra-array replicates to increase the statistical confidence and to compensate for potential positional effects. As a result the raw data files contain a total of 7 data points for each miRNA. The intensities of blank probes which consist only of one single “T” nucleotide are used for background corrections. Spike-in controls for the labeling efficiency are also present. In order to correctly control the hybridization process as well as positioning features, additional hybridization controls are added to the array template. Blank, labeling control and hybridization control probes are not included in the data analysis.

The light-activated *in-situ* oligonucleotide synthesis using a digital micromirror device was performed within the Geniom One instrument on an activated three-dimensional reaction carrier consisting of a glass-silicon-glass sandwich. Using standard DNA synthesis reagents and 3^′^-phosphoramidites carrying a 5^′^-photolabile protective group, oligonucleotides were synthesized in parallel in eight individual microchannels of one biochip. Prior to synthesis, the glass surface was activated by coating with a spacer to facilitate probe-target interaction and to avoid probe-probe interference [32].

For each array the RNA was suspended in Febit’s proprietary miRNA Hybridization Buffer (25ul per array). Hybridization was carried out automatically for 16 h at 42°C using the Geniom RT-Analyzer. In the next step the biochip was given a stringent wash. Following the labeling procedure, the microfluidic-based primer extension assay was applied [33]. This assay utilizes the bound miRNAs as a primer for an enzymatic elongation with labeled nucleotides. The elongation was carried out with Klenow Fragment and biotinylated nucleotides at 37°C for 15 minutes. Finally, the biochip was washed automatically.

For maximum sensitivity, biotin was used and detected with streptavidin-phycoerythrin (SAPE), in combination with febit’s consecutive Signal Enhancement (CSE) procedure (Febit). For a more detailed description please the read febit protocol 010 from Febit (reference). The feature recognition (using Cy3 filter set) and signal calculation were done automatically within milliseconds. The Geniom Technology showed accurate detection of miRNA profiles. The data correlated well with qPCR data. There was no photo bleaching which enabled repeated measurements and multiple detection of each Biochip.

### Bioinformatics analysis

The bioinformatics analysis starts with a summary of the measured data. Thereafter, spatial effects on the chip are investigated and corrected. Then, the intensity value distribution of raw data is analyzed and normalized. To evaluate the distribution of signal on the biochips, we computed a spatial distribution plot. Such plots show the reactivity of each transcript of the 8 arrays at the current position of each transcript. To account for variation between the hybridized arrays normalization is essential. For miRNAs, the VSN (variance stabilizing normalization) may outperform other approaches such as the quantile normalization. Applying quantile normalization, it is assumed that all arrays should show exactly the same distribution. However, especially in the case of miRNA, the underlying assumptions may not hold. In contrast, VSN is derived from a model of the variance-versus-mean dependence. While the variance of transcripts should be independent of their mean, measured data often show a quadratic dependency. This quadratic dependency can also be detected in the measured data.

Higher-level bioinformatics analyses, including correlation analysis, scatter plots, cluster analysis, venn diagrams, MA plots, principal component analysis, variance-related boxplots and so on, are executed. These analyses may help enhance our understanding to understand the principles of the applied approaches and may facilitate the interpretation of the results.

### RT-PCR

The RNA were extracted from 5 adult male Sprague-Dawley rats, we got the tissue samples of Hip and MrD in brain using the same way showed as above. A quantitative PCR was performed using a miScript SYBR-Green PCR Kit (Qiagen) following the manufacturer’s protocol. All qRT-PCR assays were done in triplicate for each sample. Briefly, for each miRNA to be assayed, 20 ng of total RNA was converted to cDNA using the miRNA-specific Taqman primer. The resulting cDNA was diluted 1:2 prior to performing qRT-PCR. The small nuclear RNA U6 was used as the normalization control. The 11 aberrantly expressed miRNA expression level was calculated with the CT method using the ABI 7300 Sequence Detection System (Applied Biosystems, Foster City, CA, USA).

### Statistical analysis

Results are presented as the mean ± standard deviation. Statistical analyses were carried out using spss13.0 software. The comparison of miRNA expression examed by RT-PCR between hippocampus and the Marginal division (MrD) was carried out using independent t-test. P<0.05 was considered statistically significant.

## Results

### miRNA array data

The miRNA expression profiles derived from different brain tissues were analyzed using oligo-microarray. For the detection of differentially regulated miRNAs, we focused on the following comparisons: LZ1 vs. LZ2, LZ3 vs. LZ4 and LZ5 vs. LZ6. Properties of the raw data are presented in Table 2. As the tables show, we have only about 0.04% flagged values. The mean background varied between the Biochips indicating that a background correction of the data is appropriate (details on the background correction are not shown).

**Table 2:** Summary on raw data of miRNA array

	**LZ1**	**LZ2**	**LZ3**	**LZ4**	**LZ5**	**LZ6**
Mean intensity	720	612	530	749	599	1168
sd intensity	1572	1398	811	759	1085	3182
Mean bg. int.	256	240	288	512	352	248
sd bg. int.	57	40	69	117	74	54
Present calls	2462	2380	1345	856	1036	3395
Present calls%	42	40	23	14	17	58
Flagged	88	130	322	63	180	14

### miRNA differential expression between Hip and MrD

To investigate the differential expression of miRNAs, array-based miRNA profiling of rat Hip and the MrD was performed. In each specimen (rat1, rat2 and rat3), we generated a short-list of the miRNAs showing greatest up- or down-regulation. The following table shows the 30 probes with the largest changes in expression (detected by highest absolute value of log fold changes) for the above comparisons (Additional file 1: Table S3).

In these most deregulated expression of miRNAs, we identified eleven miRNAs which occurred high differential expression in more than one rat simultaneously (gray lines in Additional file 1: Table S3). Hence, miR-383 was differentially expressed in three rats, and let-7d*, miR-181b, miR-187, miR-195, miR-214, miR-382, miR-411, miR-466b, miR-592, miR-1224 were differentially expressed in two rats at least (Additional file 2: Table S4). Beside miR-382 and miR-411 which were up-regulated in one rat and down-regulated in another, the other nine miRNAs kept the uniform regulated direction (up or down) between different rats (Additional file 2: Table S4).

### RT-PCR

To validate the miRNA microarray results, 11 differently expressed miRNAs was analyzed by real-time RT-PCR. Every miRNA has 3 CT values, the mean CT values were showed in the Table 3. We found that except miR-383 and let-7d*, the other 9 miRNAs were significantly higher expressed in the MrD. The RT-PCR results were partly in concordance with the miRNA microarray analysis results.

**Table 3:** The results of RT-PCR of 11 aberrantly expressed miRNAs

	**The mean CT value of Hip**	**The mean CT value of MrD**	**p-value**
rno-let-7d*	19.16±0.05	19.23±0.14	0.63
rno-miR-181b	16.68±0.05	17.62±0.19	0.00
rno-miR-187	25.31±0.08	26.84±0.19	0.00
rno-miR-195	23.11±0.08	24.34±0.14	0.00
rno-miR-214	22.47±0.10	23.49±0.20	0.00
rno-miR-382	24.88±0.18	26.04±0.17	0.00
rno-miR-383	24.06±0.03	24.36±0.14	0.06
rno-miR-411	23.94±0.21	26.39±0.33	0.00
rno-miR-466b	18.96±0.08	19.50±0.08	0.00
rno-miR-592	24.38±0.10	25.45±0.08	0.00
rno-miR-1224	21.97±0.21	22.94±0.24	0.01

## Discussion

The MrD of the neostriatum is a flat, pan-shaped structure consisting of dorsoventral spindle-shaped neurons arranged in a parallel formation, located at the caudomedial margin of the neostriatum and surrounding the dostrolateral border of the globus pallidus (GP), and distinct from other parts of the striatum in the mammalian brains. Lesion and Y-maze tests and assessment of c-Fos expression and Patch clamp analysis showed that the MrD contributes to the learning and memory function[23-30]. FMRI investigations and clinical case reports verified the contribution of the MrD to digital working and mathematical calculating memory in the human brain[34-36]. All these early experiments indicate that MrD is likely to be an important subcortical center of learning and memory based on its position, its advanced development in higher mammalian brains, its abundant blood supply and the diverse connections with other memory-related structures. Moreover, miRNAs are particularly abundant in neurons, and together with the fact that a given miRNA usually regulates the expression of hundreds of target mRNAs, neuronal miRNA pathways create an extremely powerful mechanism for dynamically adjusting the protein content of neuronal compartments without the need for new gene transcription. MiRNAs likely have a big impact on higher cognitive function including learning and memory [10,37,38]. It has been speculated that miRNA expression differing from brain region to region, may reflect brain region-specific miRNA expression patterns with a corresponding role in each brain area [39]. Recently, Juhila et al. compared miRNA expression between hippocampus and frontal cortex, and confirmed this hypothesis finding that miRNA expression in the hippocampus was extremely different from that of frontal cortex [40]. Based on the above discoveries, this research was aimed at finding those miRNAs that were differentially expressed between MrD and Hip, the well established memory related structures in the brain.

Although miRNAs have been implicated in several important biological functions in the CNS including neurogenesis [41], dendrite formation [42], brain morphogenesis [43], neural plasticity [44], and silencing of non-neuronal transcripts [45,46], far less information is available on miRNA expression patterns of neostriatum in anatomically regional differences. In this study, a miRNA array with 357 known rodent miRNA probes (15 replicates per array) was applied to sample the differential expression of miRNAs in Hip and the MrD of the rat brain. In the results, the most dysregulated 30 probes were identified in each rat. A total of 78 miRNAs were listed in Additional file 1: Table S3 and 11 miRNAs were differentially expressed in more than one rat (gray line in Additional file 1: Table S3). Although the most upregulated miRNAs in three rats were miR-383, miR-451 and miR-219-5p (with logs [FC]: -2.15, -2.21, -2.60) respectively, we wanted to focus on miRNAs that were altered in different rats simultaneously. Notably, miR-383 was not only most upregulated in rat1, but was also high in rat2 and rat3. The other ten miRNAs, up-regulated or down-regulated, were found to be high differentially affected in two different sample rats (Additional file 2: Table S4).

The importance of miRNAs in the nervous system was first described in Danio rerio (zebrafish) in which a mutation in dicer1 led to failure to produce mature miRNAs and resulted in gross morphological defects in the nervous system [47]. Effects involving regulation of specific miRNAs in neurons have been found in a number of organisms including mammals. For example, during neurogenesis, the levels of both miR-124 and miR-9 are greatly increased, and both of them were indicated involving in neuronal differentiation in vitro experiments [44,48]. Deep sequencing of miRNAs derived from tissues and cell lines have revealed these and other miRNAs are restricted to the CNS [46]. Definitive proof of the role of miR-124 in neurogenesis has now been achieved *in vivo*, revealing its critical role in the differentiation of neurons from neural precursors [49]. In addition to differentiation of neurons, miRNAs have been shown to affect crucial aspects of neurons. For example, neurite outgrowth is regulated by miR-132 [50]. Furthermore, one crucial functional aspect of neurons, the synapse, is under miRNAs control. In the hippocampus, miR-134 regulates the size of dendritic spines, sites of synaptic transmission [42]. Further linking of miRNAs to synaptic changes and the implications of such in brain development and plasticity was the recent demonstration that miR-138 controls dendritic spine morphogenesis [51]. The expression of miRNAs mentioned above was not significantly deregulated in our study, though they were verified to be more important in development, differentiation and function of CNS.

Rno-miR-383, the most interesting one of these miRNAs, is significantly up-regulated in Hip in all three rats. Its precursor possesses 73nt stem loop construction, and the mature sequence is 5^′^-cagaucagaaggugacugugg-3^′^. After bioinformatics analysis, there are 770 predicted target sites in *rattus norvegicu* genome (http://mirnamap.mbc.nctu.edu.tw/index.php). There are 17 members from different species that have been sequenced in the mir-383 family, including: bta, cfa, eca, gga, hsa, mdo, mml, mmu, oan, ppy, ptr, rno, ssc, tgu, xtr, aca, sha (http://www.mirbase.org/cgi-bin/mirna_summary.pl?fam=MIPF0000137). Experimentally, Lian et al [52] have reported that the expression of hsa-miR-383 is altered in testicular itssues of human patients, and they consider that a potential target of miR-383 may be GADD45G. GADD45G can induce apoptosis and inhibit cell growth in response to stress shock. Abnormal expressions of these proteins may have a significant impact on male infertility [53,54]. However, no evidence demonstrates how rno-miR-383 is involved into complicated regulatory net-works in CNS, especially in MrD.

Beside rno-miR-383, there are also 10 miRNAs differentially expressed in only two rats in this study. In these 10 deregulated miRNAs, seven (let-7d*, miR-181b, miR-187, miR-214, miR-466b, miR-592, miR-1224) are up-regulated in two rats, and one (miR-195) is down-regulated. The last two investigated, miR-382 and miR-411, were up-regulated in one rat and down-regulated in another. It is possible to speculate that non tissue-specific expression may be a reason for this unexpected expression pattern of some miRNAs, and meanwhile they were also expressed in many different tissues in random manner. The other reasons may be the intrinsic variability of these miRNAs in this brain region or the effect correlate with some behavioral variability. Except for let-7d*, the RT-PCR results showed the same trend with that of microarray, which means that it validated the expression patterns we found in our microarray experiments.

Our previous study has shown that the MrD-NBM-hippocampus circuit may play an important role in modulating learning and memory [23-30]. Tissue-specific miRNAs expression patterns as determined in this study can make important contributions to understanding regulatory expression networks. Although difficult for small RNAs, there is no substitute for *in situ* hybridization studies to follow up on various experimental findings in order to assess the regions and cell types responsible for distinct miRNA expression patterns. Meanwhile, differential expression of identified miRNAs can be then assessed in models of disease or in diseased tissue itself. In summary, the study on miRNAs, their mRNA targets, and resulting changes in protein products will continue to be an exciting field of research, leading to a greater understanding of the regulatory effects of the miRNA.

## Conclusion

This is the first evidence for the comparison of miRNA expression profiles between hippocampus and the MrD of the striatum in rat brain. In dysregulated miRNAs, let-7d*, miR-181b, miR-187, miR-214, miR-383, miR-466b, miR-592, miR-1224 are significantly overexpressed in MrD compared to hippocampus, and miR-195 is downexpressed. More study is required to clarify the precise contributions of miRNAs in the MrD and the hippocampal circuit.

## Competing interests

We declare that we have no competing interests.

## Authors’ contributions

SYS designed the research, directed and organized the experiments and drafted the manuscript; BW organized the experiments; DQ participated the microarray experiments and jointed in the revising of manuscript. YCC and RB carried out the RT-PCR, CCZ did the date analysis, QYZ and YJ provided valuable opinions for revising the manuscript. All authors read and approved the final manuscript.

## Supplementary Material

Additional file 1: Table S3 Differentially regulated miRNAs expression between Hip and MrD. Click here for file

Additional file 2: Table S4 The details of different expression of 11 miRNAs mentioned above. Click here for file
