# Supplementary material for: Comparison of microRNA expression in hippocampus and the marginal division (MrD) of the neostriatum in rats
Source: J Biomed Sci. 2013 Feb 20;20(1):9. doi: 10.1186/1423-0127-20-9 (PMC3615960; doi:10.1186/1423-0127-20-9)
Supplement: Additional file 1: Table S3 — Differentially regulated miRNAs expression between Hip and MrD. [file 1423-0127-20-9-S1.doc]

**Table 3:** Differentially regulated miRNAs expression between Hip and MrD

| Rat 1 Rat2 Rat3  M1 M2 qM12 logqM12 M3 M4 qM34 logqM34 M5 M6 qM56 logqM56 |
| --- |
| | rno-let-7a |  |  |  |  |  |  |  |  | 7.69 | 9.23 | 0.21 | -1.53 | | --- | --- | --- | --- | --- | --- | --- | --- | --- | --- | --- | --- | --- | | rno-let-7b |  |  |  |  | 7.99 | 6.89 | 3.00 | 1.10 |  |  |  |  | | rno-let-7c |  |  |  |  | 8.36 | 7.06 | 3.67 | 1.30 |  |  |  |  | | rno-let-7d* |  |  |  |  | 5.27 | 6.36 | 0.34 | -1.08 | 5.21 | 6.66 | 0.23 | -1.45 | | rno-let-7e |  |  |  |  |  |  |  |  | 7.28 | 8.88 | 0.20 | -1.59 | | rno-let-7f |  |  |  |  | 7.05 | 5.43 | 5.05 | 1.62 |  |  |  |  | | rno-let-7i |  |  |  |  | 5.77 | 4.42 | 3.89 | 1.36 |  |  |  |  | | rno-let-7i* | 8.24 | 6.79 | 4.30 | 1.46 |  |  |  |  |  |  |  |  | | rno-miR-9 | 9.26 | 7.62 | 5.18 | 1.64 |  |  |  |  |  |  |  |  | | rno-miR-10a-3p |  |  |  |  |  |  |  |  | 6.57 | 4.65 | 6.97 | 1.92 | | rno-miR-23a* | 4.63 | 6.12 | 0.22 | -1.49 |  |  |  |  |  |  |  |  | | rno-miR-29a |  |  |  |  |  |  |  |  | 10.8 | 12.4 | 0.20 | -1.59 | | rno-miR-30c-2* |  |  |  |  |  |  |  |  | 3.62 | 5.49 | 0.15 | -1.87 | | rno-miR-32 |  |  |  |  |  |  |  |  | 7.06 | 5.39 | 5.28 | 1.66 | | rno-miR-34a |  |  |  |  | 5.88 | 4.83 | 2.87 | 1.05 |  |  |  |  | | rno-miR-93 |  |  |  |  | 6.75 | 5.62 | 3.10 | 1.13 |  |  |  |  | | rno-miR-96 | 6.64 | 4.67 | 7.17 | 1.97 |  |  |  |  |  |  |  |  | | rno-miR-99a | 10.28 | 9.01 | 3.55 | 1.27 |  |  |  |  |  |  |  |  | | rno-miR-100 | 9.53 | 8.24 | 3.62 | 1.29 |  |  |  |  |  |  |  |  | | rno-miR-125b-3p |  |  |  |  | 3.55 | 5.62 | 0.13 | -2.05 |  |  |  |  | | rno-miR-125b-5p |  |  |  |  | 11.14 | 9.98 | 3.20 | 1.16 |  |  |  |  | | rno-miR-129 |  |  |  |  | 5.99 | 7.21 | 0.29 | -1.22 |  |  |  |  | | rno-miR-132 |  |  |  |  |  |  |  |  | 10.4 | 12.1 | 0.19 | -1.64 | | rno-miR-136 |  |  |  |  | 3.55 | 5.04 | 0.23 | -1.48 |  |  |  |  | | rno-miR-137 | 7.22 | 5.58 | 5.18 | 1.64 |  |  |  |  |  |  |  |  | | rno-miR-138 | 10.28 | 11.59 | 0.27 | -1.30 |  |  |  |  |  |  |  |  | | rno-miR-139-5p | 8.67 | 10.18 | 0.22 | -1.52 |  |  |  |  |  |  |  |  | | rno-miR-140 |  |  |  |  | 3.78 | 5.94 | 0.11 | -2.17 |  |  |  |  | | rno-miR-144 |  |  |  |  | 6.45 | 5.24 | 3.35 | 1.21 |  |  |  |  | | rno-miR-150 |  |  |  |  |  |  |  |  | 7.12 | 8.68 | 0.21 | -1.56 | | rno-miR-181b |  |  |  |  | 6.09 | 7.35 | 0.28 | -1.26 | 6.87 | 8.29 | 0.24 | -1.42 | | rno-miR-187 | 5.22 | 6.89 | 0.19 | -1.67 |  |  |  |  | 3.50 | 5.68 | 0.11 | -2.17 | | rno-miR-195 | 10.91 | 9.40 | 4.57 | 1.52 |  |  |  |  | 11.1 | 9.62 | 4.42 | 1.49 | | rno-miR-199a-3p | 5.90 | 4.67 | 3.43 | 1.23 |  |  |  |  |  |  |  |  | | rno-miR-199a-5p |  |  |  |  |  |  |  |  | 6.21 | 4.54 | 5.27 | 1.66 | | rno-miR-200b | 4.63 | 6.67 | 0.13 | -2.04 |  |  |  |  |  |  |  |  | | rno-miR-200c | 5.22 | 6.79 | 0.21 | -1.56 |  |  |  |  |  |  |  |  | | rno-miR-202 | 5.75 | 4.43 | 3.37 | 1.31 |  |  |  |  |  |  |  |  | | rno-miR-206 |  |  |  |  |  |  |  |  | 4.00 | 5.59 | 0.20 | -1.59 | | rno-miR-214 | 9.88 | 11.12 | 0.29 | -1.24 |  |  |  |  | 9.74 | 11.2 | 0.23 | -1.46 | | rno-miR-218 | 9.60 | 8.36 | 3.47 | 1.24 |  |  |  |  |  |  |  |  | | rno-miR-219-5p |  |  |  |  |  |  |  |  | 4.00 | 6.61 | 0.07 | -2.60 | | rno-miR-221 |  |  |  |  | 6.09 | 7.42 | 0.26 | -1.33 |  |  |  |  | | rno-miR-291a-3p |  |  |  |  | 5.53 | 3.85 | 5.42 | 1.69 |  |  |  |  | | rno-miR-296 |  |  |  |  |  |  |  |  | 6.21 | 7.75 | 0.21 | -1.55 | | rno-miR-327 | 5.14 | 6.99 | 0.20 | -1.59 |  |  |  |  |  |  |  |  | | rno-miR-338 |  |  |  |  |  |  |  |  | 7.43 | 9.02 | 0.20 | 1.59 | | rno-miR-340-3p |  |  |  |  | 3.78 | 4.83 | 0.35 | -1.05 |  |  |  |  | | rno-miR-363 |  |  |  |  | 5.41 | 6.59 | 0.31 | -1.18 |  |  |  |  | | rno-miR-369-5p | 6.24 | 5.14 | 3.61 | 1.28 |  |  |  |  |  |  |  |  | | rno-miR-370 |  |  |  |  |  |  |  |  | 4.00 | 6.30 | 0.10 | -2.30 | | rno-miR-374 | 7.08 | 5.87 | 3.35 | 1.21 |  |  |  |  |  |  |  |  | | rno-miR-376a |  |  |  |  |  |  |  |  | 7.12 | 8.56 | 0.24 | -1.44 | | rno-miR-382 |  |  |  |  | 5.88 | 4.83 | 2.87 | 1.05 | 5.50 | 7.36 | 0.16 | -1.86 | | rno-miR-383 | 6.64 | 8.79 | 0.12 | -2.15 | 7.26 | 8.57 | 0.27 | -1.30 | 7.00 | 8.90 | 0.15 | -1.90 | | rno-miR-384-3p | 5.90 | 4.67 | 3.43 | 1.23 |  |  |  |  |  |  |  |  | | rno-miR-409-3p |  |  |  |  | 5.66 | 4.02 | 5.12 | 1.63 |  |  |  |  | | rno-miR-411 |  |  |  |  | 6.45 | 4.83 | 5.04 | 1.62 | 5.21 | 6.82 | 0.20 | -1.61 | | rno-miR-425 |  |  |  |  | 7.57 | 6.23 | 3.82 | 1.34 |  |  |  |  | | rno-miR-449a | 3.80 | 5.02 | 0.29 | -1.22 |  |  |  |  |  |  |  |  | | rno-miR-451 |  |  |  |  | 4.02 | 6.23 | 0.11 | -2.21 |  |  |  |  | | rno-miR-466b |  |  |  |  | 3.78 | 5.79 | 0.13 | -2.01 | 4.44 | 6.23 | 0.17 | -1.79 | | rno-miR-490 | 6.64 | 5.14 | 4.47 | 1.50 |  |  |  |  |  |  |  |  | | rno-miR-497 | 9.35 | 7.85 | 4.55 | 1.49 |  |  |  |  |  |  |  |  | | rno-miR-505 |  |  |  |  | 5.14 | 3.58 | 3.64 | 1.29 |  |  |  |  | | rno-miR-541 |  |  |  |  |  |  |  |  | 4.90 | 6.37 | 0.23 | -1.46 | | rno-miR-543* |  |  |  |  |  |  |  |  | 3.74 | 5.93 | 0.11 | -2.19 | | rno-miR-551b | 7.42 | 5.37 | 7.79 | 2.05 |  |  |  |  |  |  |  |  | | rno-miR-592 |  |  |  |  | 5.14 | 6.79 | 0.19 | -1.66 | 3.50 | 5.59 | 0.12 | -2.08 | | rno-miR-598-3p |  |  |  |  |  |  |  |  | 4.75 | 6.96 | 0.11 | -2.21 | | rno-miR-672 |  |  |  |  |  |  |  |  | 3.50 | 5.39 | 0.15 | -1.89 | | rno-miR-685 | 6.04 | 7.34 | 0.28 | -1.29 |  |  |  |  |  |  |  |  | | rno-miR-711 |  |  |  |  | 5.88 | 7.28 | 0.25 | -1.40 |  |  |  |  | | rno-miR-760-3p | 5.09 | 7.55 | 0.19 | -1.65 |  |  |  |  |  |  |  |  | | rno-miR-872* |  |  |  |  | 5.88 | 4.62 | 3.54 | 1.26 |  |  |  |  | | rno-miR-877 | 7.79 | 9.30 | 0.29 | -1.24 |  |  |  |  |  |  |  |  | | rno-miR-880 |  |  |  |  | 6.09 | 4.83 | 3.53 | 1.26 |  |  |  |  | | rno-miR-1224 | 9.06 | 10.32 | 0.28 | -1.27 |  |  |  |  | 8.53 | 10.3 | 0.16 | -1.84 | |
| Mi represents median of group i (i=LZ1, LZ2, LZ3, LZ4, LZ5, LZ6). qM and logqM represent median fold change (FC) and log fold change. During the normalization of the miRNA data set we are using VSN (Variance Stabilisation normalization) method. Therefore it is needed to de-logarithmize before we calculate the qmedian (qmedian=e^(M1-M2)^ , where M1=median of LZ1 and M2=median of LZ2 ). Finally, logqmedian is with basis e (natural logarthm where e=2.71828). |
