# Supplementary material for: Comparison of microRNA expression in hippocampus and the marginal division (MrD) of the neostriatum in rats
Source: J Biomed Sci. 2013 Feb 20;20(1):9. doi: 10.1186/1423-0127-20-9 (PMC3615960; doi:10.1186/1423-0127-20-9)
Supplement: Additional file 2: Table S4 — The details of different expression of 11 miRNAs mentioned above. [file 1423-0127-20-9-S2.doc]

**Table 4:** The details of different expression of 11 miRNAs mentioned above

| fold change up- or down- expression  (loge) regulated position |
| --- |
| | rno-let-7d* | -1.08 | up | LZ3 vs LZ4 (rat2) | | --- | --- | --- | --- | |  | -1.45 | up | LZ5 vs LZ6 (rat3) | | rno-miR-181b | -1.26 | up | LZ3 vs LZ4 (rat2) | |  | -1.42 | up | LZ5 vs LZ6 (rat3) | | rno-miR-187 | -1.67 | up | LZ1 vs LZ2 (rat1) | |  | -2.17 | up | LZ5 vs LZ6 (rat3) | | rno-miR-195 | 1.52 | down | LZ1 vs LZ2 (rat1) | |  | 1.49 | down | LZ5 vs LZ6 (rat3) | | rno-miR-214 | -1.24 | up | LZ1 vs LZ2 (rat1) | |  | -1.46 | up | LZ5 vs LZ6 (rat3) | | rno-miR-382 | 1.05 | down | LZ3 vs LZ4 (rat2) | |  | -1.86 | up | LZ5 vs LZ6 (rat3) | | rno-miR-383 | -2.15 | up | LZ1 vs LZ2 (rat1) | |  | -1.30 | up | LZ3 vs LZ4 (rat2) | |  | -1.90 | up | LZ5 vs LZ6 (rat3) | | rno-miR-411 | 1.62 | down | LZ3 vs LZ4 (rat2) | |  | -1.61 | up | LZ5 vs LZ6 (rat3) | | rno-miR-466b | -2.01 | up | LZ3 vs LZ4 (rat2) | |  | -1.79 | up | LZ5 vs LZ6 (rat3) | | rno-miR-592 | -1.66 | up | LZ3 vs LZ4 (rat2) | |  | -2.08 | up | LZ5 vs LZ6 (rat3) | | rno-miR-1224 | -1.27 | up | LZ1 vs LZ2 (rat1) | |  | -1.84 | up | LZ5 vs LZ6 (rat3) | |
